# Supplementary material for: Multi‐dataset identification of innovative feature genes and molecular mechanisms in keratoconus
Source: J Cell Mol Med. 2024 Sep 19;28(18):e70079. doi: 10.1111/jcmm.70079 (PMC11412914; doi:10.1111/jcmm.70079)
Supplement: Supplementary file 2 — Material S2. [file JCMM-28-e70079-s002.zip › 7.GSEA analysis/3.DBNDD1/KEGG_DILATED_CARDIOMYOPATHY.html]

Details for gene set KEGG\_DILATED\_CARDIOMYOPATHY[GSEA]

|  || Dataset | DBNDD1.group.cls#h\_versus\_l |
| Phenotype | group.cls#h\_versus\_l |
| Upregulated in class | l |
| GeneSet | KEGG\_DILATED\_CARDIOMYOPATHY |
| Enrichment Score (ES) | -0.35370952 |
| Normalized Enrichment Score (NES) | -1.2783369 |
| Nominal p-value | 0.11309524 |
| FDR q-value | 0.71373856 |
| FWER p-Value | 0.971 |
Table: GSEA Results Summary

  

Fig 1: Enrichment plot: KEGG\_DILATED\_CARDIOMYOPATHY      
 Profile of the Running ES Score & Positions of GeneSet Members on the Rank Ordered List

  

| SYMBOL | TITLE | RANK IN GENE LIST | RANK METRIC SCORE | RUNNING ES | CORE ENRICHMENT || 1 | TNNT2 | na | 514 | 0.448 | 0.0187 | No |
| 2 | CACNB1 | na | 587 | 0.431 | 0.0489 | No |
| 3 | TNNI3 | na | 657 | 0.420 | 0.0783 | No |
| 4 | CACNA1F | na | 1766 | 0.316 | 0.0699 | No |
| 5 | PLN | na | 1912 | 0.306 | 0.0886 | No |
| 6 | ACTC1 | na | 2612 | 0.267 | 0.0885 | No |
| 7 | CACNG5 | na | 2873 | 0.257 | 0.1002 | No |
| 8 | CACNG7 | na | 4144 | 0.209 | 0.0791 | No |
| 9 | SGCD | na | 4837 | 0.185 | 0.0730 | No |
| 10 | TPM2 | na | 5034 | 0.180 | 0.0808 | No |
| 11 | DAG1 | na | 5307 | 0.170 | 0.0856 | No |
| 12 | ADCY7 | na | 6033 | 0.150 | 0.0759 | No |
| 13 | ADCY9 | na | 6468 | 0.137 | 0.0736 | No |
| 14 | ADCY6 | na | 6632 | 0.133 | 0.0789 | No |
| 15 | ITGA4 | na | 7459 | 0.114 | 0.0636 | No |
| 16 | TNNC1 | na | 7636 | 0.111 | 0.0668 | No |
| 17 | EMD | na | 7789 | 0.108 | 0.0705 | No |
| 18 | TTN | na | 7964 | 0.104 | 0.0733 | No |
| 19 | CACNG1 | na | 8316 | 0.098 | 0.0705 | No |
| 20 | ADCY4 | na | 8335 | 0.097 | 0.0773 | No |
| 21 | ADCY5 | na | 8470 | 0.095 | 0.0805 | No |
| 22 | LAMA2 | na | 9618 | 0.077 | 0.0531 | No |
| 23 | ITGB7 | na | 9817 | 0.074 | 0.0529 | No |
| 24 | CACNA2D3 | na | 10208 | 0.068 | 0.0468 | No |
| 25 | IGF1 | na | 10555 | 0.064 | 0.0416 | No |
| 26 | ATP2A2 | na | 11124 | 0.057 | 0.0294 | No |
| 27 | CACNA1D | na | 13311 | 0.036 | -0.0310 | No |
| 28 | DES | na | 13515 | 0.034 | -0.0343 | No |
| 29 | TGFB3 | na | 13624 | 0.033 | -0.0350 | No |
| 30 | CACNB2 | na | 14012 | 0.030 | -0.0439 | No |
| 31 | MYBPC3 | na | 14239 | 0.028 | -0.0483 | No |
| 32 | TPM4 | na | 14283 | 0.028 | -0.0475 | No |
| 33 | ACTB | na | 14636 | 0.025 | -0.0558 | No |
| 34 | CACNG8 | na | 15428 | 0.020 | -0.0771 | No |
| 35 | PRKX | na | 15799 | 0.017 | -0.0865 | No |
| 36 | DMD | na | 15967 | 0.016 | -0.0901 | No |
| 37 | ITGA2B | na | 16436 | 0.013 | -0.1027 | No |
| 38 | TPM1 | na | 17928 | 0.003 | -0.1455 | No |
| 39 | ACTG1 | na | 18138 | 0.002 | -0.1514 | No |
| 40 | ADCY2 | na | 19167 | -0.006 | -0.1806 | No |
| 41 | TGFB2 | na | 19303 | -0.007 | -0.1840 | No |
| 42 | LMNA | na | 20345 | -0.016 | -0.2128 | No |
| 43 | ITGB4 | na | 20511 | -0.017 | -0.2163 | No |
| 44 | PRKACA | na | 20814 | -0.020 | -0.2235 | No |
| 45 | ITGA9 | na | 21410 | -0.026 | -0.2387 | No |
| 46 | ITGB5 | na | 21935 | -0.031 | -0.2515 | No |
| 47 | GNAS | na | 22042 | -0.033 | -0.2521 | No |
| 48 | ADRB1 | na | 22618 | -0.039 | -0.2659 | No |
| 49 | TPM3 | na | 23026 | -0.043 | -0.2744 | No |
| 50 | ADCY8 | na | 23069 | -0.044 | -0.2723 | No |
| 51 | CACNA1C | na | 23124 | -0.045 | -0.2705 | No |
| 52 | ADCY3 | na | 23170 | -0.045 | -0.2684 | No |
| 53 | MYH7 | na | 23841 | -0.054 | -0.2837 | No |
| 54 | ITGA3 | na | 24015 | -0.056 | -0.2845 | No |
| 55 | MYL2 | na | 25552 | -0.079 | -0.3229 | No |
| 56 | MYL3 | na | 25616 | -0.081 | -0.3187 | No |
| 57 | ITGAV | na | 25782 | -0.083 | -0.3172 | No |
| 58 | ITGA11 | na | 26521 | -0.098 | -0.3312 | No |
| 59 | PRKACB | na | 26730 | -0.102 | -0.3295 | No |
| 60 | ITGA2 | na | 26752 | -0.102 | -0.3225 | No |
| 61 | TNF | na | 27164 | -0.111 | -0.3260 | No |
| 62 | PRKACG | na | 28125 | -0.134 | -0.3437 | Yes |
| 63 | CACNG4 | na | 28414 | -0.141 | -0.3415 | Yes |
| 64 | ITGA6 | na | 28732 | -0.150 | -0.3394 | Yes |
| 65 | ITGA7 | na | 29075 | -0.159 | -0.3374 | Yes |
| 66 | ITGB1 | na | 29102 | -0.160 | -0.3262 | Yes |
| 67 | SGCA | na | 29145 | -0.161 | -0.3154 | Yes |
| 68 | SGCB | na | 29234 | -0.164 | -0.3056 | Yes |
| 69 | CACNB3 | na | 29250 | -0.164 | -0.2938 | Yes |
| 70 | CACNA2D4 | na | 29251 | -0.164 | -0.2815 | Yes |
| 71 | ITGB3 | na | 29286 | -0.165 | -0.2701 | Yes |
| 72 | MYH6 | na | 29406 | -0.169 | -0.2609 | Yes |
| 73 | ITGB6 | na | 29433 | -0.170 | -0.2489 | Yes |
| 74 | SGCG | na | 29686 | -0.179 | -0.2428 | Yes |
| 75 | ADCY1 | na | 29881 | -0.185 | -0.2346 | Yes |
| 76 | ITGB8 | na | 30390 | -0.200 | -0.2343 | Yes |
| 77 | TGFB1 | na | 30914 | -0.218 | -0.2331 | Yes |
| 78 | ITGA1 | na | 31047 | -0.222 | -0.2203 | Yes |
| 79 | CACNG6 | na | 31154 | -0.226 | -0.2064 | Yes |
| 80 | SLC8A1 | na | 32046 | -0.266 | -0.2122 | Yes |
| 81 | RYR2 | na | 32172 | -0.274 | -0.1953 | Yes |
| 82 | ITGA10 | na | 32340 | -0.284 | -0.1789 | Yes |
| 83 | CACNA2D2 | na | 32648 | -0.304 | -0.1650 | Yes |
| 84 | CACNG2 | na | 33176 | -0.343 | -0.1546 | Yes |
| 85 | CACNA2D1 | na | 33832 | -0.408 | -0.1430 | Yes |
| 86 | CACNB4 | na | 33927 | -0.420 | -0.1143 | Yes |
| 87 | ITGA5 | na | 34082 | -0.440 | -0.0858 | Yes |
| 88 | ITGA8 | na | 34117 | -0.445 | -0.0535 | Yes |
| 89 | CACNA1S | na | 34256 | -0.474 | -0.0220 | Yes |
| 90 | CACNG3 | na | 34262 | -0.475 | 0.0135 | Yes |
Table: GSEA details [plain text format]

  

Fig 2: KEGG\_DILATED\_CARDIOMYOPATHY      
 Blue-Pink O' Gram in the Space of the Analyzed GeneSet

  

Fig 3: KEGG\_DILATED\_CARDIOMYOPATHY: Random ES distribution      
 Gene set null distribution of ES for **KEGG\_DILATED\_CARDIOMYOPATHY**
